# Chitosan/Polycyclodextrin (CHT/PCD)-Based Sponges Delivering VEGF to Enhance Angiogenesis for Bone Regeneration

**DOI:** 10.3390/pharmaceutics12090784

**Published:** 2020-08-19

**Authors:** Carla Palomino-Durand, Marco Lopez, Pierre Marchandise, Bernard Martel, Nicolas Blanchemain, Feng Chai

**Affiliations:** 1 U1008 Controlled Drug Delivery Systems and Biomaterials, Institut National de la Santé et de la Recherche Médicale (INSERM), Centre Hospitalier Régional Universitaire de Lille (CHU Lille), University of Lille, 59000 Lille, France; cpalominodurand@gmail.com (C.P.-D.); marco.lopez@univ-lille.fr (M.L.); nicolas.blanchemain@univ-lille.fr (N.B.); 2 ULR 4490–MABLab–Adiposité Médullaire et Os, Institut National de la Santé et de la Recherche Médicale (INSERM), Centre Hospitalier Régional Universitaire de Lille (CHU Lille), University of Lille, 59000 Lille, France; pierre.marchandise@univ-lille.fr; 3 ULR 4490–MABLab–Adiposité Médullaire et Os, Univ. Littoral Côte d’Opale, 62200 Boulogne-sur-Mer, France; 4 UMR 8207, UMET—Unité Matériaux et Transformations, Centre National de la Recherche Scientifique (CNRS), Institut National de la Recherche Agronomique (INRA), Ecole Nationale Supérieure de Chimie de Lille (ENSCL), University of Lille, 59655 Lille, France; bernard.martel@univ-lille.fr

**Keywords:** sponges, cyclodextrin polymer, chitosan, VEGF-delivery, bone tissue engineering, cell migration, cell proliferation

## Abstract

Vascularization is one of the main challenges in bone tissue engineering (BTE). In this study, vascular endothelial growth factor (VEGF), known for its angiogenic effect, was delivered by our developed sponge, derived from a polyelectrolyte complexes hydrogel between chitosan (CHT) and *anionic* cyclodextrin polymer (PCD). This sponge, as a scaffold for growth factor delivery, was formed by freeze-drying a homogeneous CHT/PCD hydrogel, and thereafter stabilized by a thermal treatment. Microstructure, water-uptake, biodegradation, mechanical properties, and cytocompatibility of sponges were assessed. VEGF-delivery following incubation in medium was then evaluated by monitoring the VEGF-release profile and its bioactivity. CHT/PCD sponge showed a porous (open porosity of 87.5%) interconnected microstructure with pores of different sizes (an average pore size of 153 μm), a slow biodegradation (12% till 21 days), a high water-uptake capacity (~600% in 2 h), an elastic property under compression (elastic modulus of compression 256 ± 4 kPa), and a good cytocompatibility in contact with osteoblast and endothelial cells. The kinetic release of VEGF was found to exert a pro-proliferation and a pro-migration effect on endothelial cells, which are two important processes during scaffold vascularization. Hence, CHT/PCD sponges were promising vehicles for the delivery of growth factors in BTE.

## 1. Introduction

Bone is a dynamic tissue with metabolic, physical, and endocrine roles in the human body [1]. Bone loss, caused by trauma or disease, is a major clinical and socio-economic problem [2,3]. Lesions greater than 3 mm, known as large bone defects, do not present a sufficient natural regeneration process and surgeons must use either a bone autograft (the gold standard) or a bone substitute to promote healing [3,4]. Nowadays, bone tissue engineering (BTE) for bone regeneration can help to overcome the drawbacks related to the use of autografts [5]. Nevertheless, large bone defects are still very difficult to treat clinically, due to the low, or lack of, blood supply (diffusion limit), which leads to the lack of nutrients and oxygen and subsequently to an implant failure (inner-graft necrosis) [6,7]. Thus, vascularization is indubitably a major challenge of BTE [7,8] and has slowed the clinical translation of engineered bone constructs.

Therefore, the innovations in vascular tissue engineering must be developed in parallel with BTE efforts. One strategy for vascularization is based on the use of angiogenic growth factors (GF) [9]. *In-situ* vascularization is established by cell invasion in response to angiogenic GF at the site of implantation. This strategy is advantageous because the new vascular network is specific to the host [10]. Vascular endothelial growth factor (VEGF), a family of homodimer proteins, is one of the most important GFs for the regulation of angiogenesis and vascular development under both physiological and pathological conditions [11]. It has been reported that VEGF participates at several stages of bone repair and regeneration, such as differentiation, proliferation, and activation of endothelial cells [12]; and the recruitment and survival of osteoblasts and osteoclasts [13], therefore, enhancing osteogenesis [14]. Numerous studies have shown the pro-angiogenic effect of VEGF in bone regeneration [15,16,17,18].

To this end, incorporation of VEGF in a scaffold increases its therapeutic effect in bone regeneration by protecting it from rapid degradation [19]. Different techniques for incorporating GFs into scaffolds have been developed in order to reach an appropriate release kinetics and stability, including physical adsorption [20,21], non-covalent and covalent immobilization [22,23], and the application of micro/nanoparticles [15,24]. Regarding the VEGF loading in literature [25,26], the physical adsorption seems to be interesting due to the avoidance of chemical modification or possible denaturation of the biomolecules [19]. 

In addition, the choice of scaffolds is very important in order to obtain a localized, sustained, and prolonged delivery of biomolecules. Hydrogels, a three-dimensional network of entangled polymeric chains swollen with water, are the biomaterials of choice for BTE. Among the different methods of hydrogel fabrication, those obtained by gas foaming, solvent casting or freeze-drying, called sponges, exhibit a mechanically stronger architecture similar to that of the extracellular matrix (ECM) and ideally serve as supports for exogenous cells or vehicles for trapping and delivering biomolecules (such as drugs, GF, and cytokines) [27,28,29] to fill large bone defects for tissue regeneration [30,31].

Hydrogels formed by polyelectrolyte complexation (PEC) of oppositely charged biopolymers, free of any chemical additives, are promising candidates for biomedical applications because they are biocompatible and eco-friendly. Among the *anionic* polyelectrolytes, the *anionic* poly(cyclodextrin/citrate) (PCD) is particularly interesting for its biocompatibility and capacity for the formation of inclusions with drugs for drug delivery application. For the *cationic* polymers, chitosan is particularly attractive because it is biosourced, biocompatible and antibacterial [32]. The combination of PCD and CHT allows the formation of PEC hydrogels. The application of these injectable CHT-PCD hydrogels for drug delivery in wound dressing [33] and tissue engineering [34] were recently reported by our team.

This work further focused on the “derivative” of CHT-PCD hydrogels, *sponges*, in order to provide sustained delivery and spatial control of bioactive VEGF in bone defects. This sponge was formed by freeze-drying a homogeneous CHT/PCD hydrogel, and thereafter stabilized by a thermal treatment. Microstructure, water-uptake, biodegradation, mechanical properties and cytocompatibility of sponges were firstly characterized. VEGF was then incorporated in the sponge, and VEGF-delivery following incubation in medium was investigated by monitoring the VEGF-release profile and its bioactivity in contact with endothelial cells.

## 2. Materials and Methods

### 2.1. Materials

Chitosan (CHT: batch STBG1894V, *M_w_* = 190 kDa, determined by gel permeation chromatography (GPC); degree of deacetylation = 73%, determined by ^1^H NMR), lactic acid (LA, >85%) and phosphate buffered saline (PBS, pH 7.4) were purchased from Sigma Aldrich (Saint Quentin, France). Water-soluble cyclodextrin polymer, poly(cyclodextrin citrate) (PCD:*M_w_* = 20 kDa, determined by size exclusion chromatography (SEC); %CD content = 58%, determined by NRM) was obtained from the reaction of esterification between β-cyclodextrin (Kleptose^®^, Roquette, Lestrem, France) and citric acid (Sigma Aldrich, Saint Quentin, France) acting as cross-linking agent and sodium hypophosphite as catalyst [34,35,36]. Recombinant human Vascular Endothelial Growth Factor A-165 (VEGF) was purchased from Promokine (Heidelberg, Germany), and enzyme-linked immunosorbent assay (ELISA) kit for VEGF was obtained from Peprotech (Neuilly-sur-Seine, France).

For the in vitro cell culture, MC3T3-E1 cells (murine pre-osteoblasts) were obtained from the American Type Culture Collection (ATCC^®^, Manassas, VA, USA) and the Human Umbilical Vein Endothelial Cells (HUVECs) isolated from the umbilical cord vein from single donors was purchased from PromoCell GmbH (Heidelberg, Germany). Ultrapure water (18.2 MΩ.cm), used for all the experiences, was produced by the water purification system EGLA VEOLIA (Purelab^®^ flex, ELGA, High Wycombe, UK).

### 2.2. Methods

#### 2.2.1. Preparation of CHT/PCD Sponges

**Figure 1:**
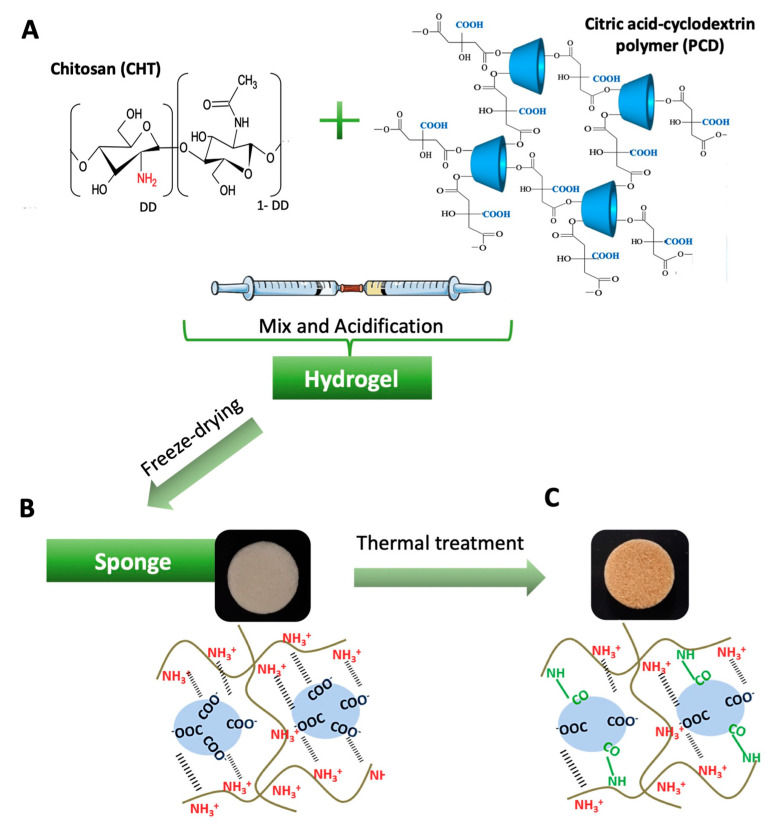
Schematic representation of the formation of chitosan/*anionic* cyclodextrin polymer (CHT/PCD) 3:3 sponge. (**A**) Chemical structure of CHT and PCD and the double-syringe system used for the obtaining of hydrogel. Macroscopic observation of sponge and the molecular interactions between CHT and PCD (**B**) after freeze-drying and (**C**) after thermal treatment.

CHT/PCD sponges were prepared by freeze-drying. First, the CHT and PCD powders (particle size < 125 µm) were co-milled at a ratio of CHT:PCD 3:3 using a mixer mill MM-40 (Retsch^®^, Haan, Germany) at 10 Hz for 3 min at room temperature (RT). Then, CHT/PCD hydrogel was obtained using a system of two interconnected syringes as described previously by Palomino-Durand et al. [34]. Briefly, co-milled CHT/PCD powders (6%*_w/v_*) and ultrapure water (93%*_v/v_*), each in a syringe (BD Luer-Lok™, Le Pont de Claix, France), were thoroughly mixed by pressing alternately on the plungers for 1 min. Afterwards, the mixture was acidified by adding lactic acid to reach a final concentration of 1%*_v/v_* in formed hydrogel, and mixed again as mentioned above for 1 min (Figure 1A). The addition of acid leads to better dissolution of CHT by the protonation of amino groups of CHT, which thereafter electrostatically interact with the carboxylate groups of PCD [33]. The obtained hydrogel was injected into a mold (12 mm diameter × 50 mm length), then frozen overnight at −20 °C and freeze-dried for 24 h at 0.06 mbar and −53 °C using the Alpha 1–2 freeze-dryer (Christ^®^, Osterode am Harz, Germany). Finally, the obtained sponges were cut into discs of different dimensions, as shown in Figure 1B, according to the need of the study, with a biopsy punch (Kai Medical, France). In order to improve structural stability of the PEC [37], CHT/PCD sponges were heat-treated at 160 °C for 90 min in a UFP600 ventilated universal oven (Memmert GmbH, Buechenbach, Germany) (Figure 1C).

#### 2.2.2. Characterization of Macroporous Hydrogels

##### Microstructure

Scanning Electron Microscopy (SEM)

The microstructure of the sponge was evaluated using SEM. The CHT/PCD sponge disks were metallized by platinum sputtering (Model 682 PECS, ADL, Binghamton, NY, USA) before observation by SEM (Hitachi S-4700, Düsseldorf, Germany) using an acceleration voltage of 5 kV and at an emission current of 10 μA.

X-Ray Microtomography (μCT) Imaging and Quantitative Analysis

A CHT/PCD sponge disk (12 mm in diameter × 5 mm thick) was scanned using a SkyScan 1172 X-ray microtomograph (Bruker microCT^®^, Kontich, Belgium). Acquisition parameters were defined according to manufacturer recommendations as follow: 40 kV, 250 µA, 675 ms exposure time, 0.1° rotation step over 180°, frame averaging 3, pixel size 3.74 µm, 4000 px by 2664 px field of view. Acquisition datasets were reconstructed with NRecon^©^ software (Bruker microCT^®^, Kontich, Belgium) according to manufacturer recommendations. Reconstructed datasets were analyzed with CTAn^©^ software (Bruker microCT^®^, Kontich, Belgium) to measure morphometric parameters (interconnectivity, porosity, and pore size) following the protocol proposed by Lopez-Heredia et al. [38].

Water Uptake

CHT/PCD sponges were weighed and placed in a vial, then a phosphate buffered saline (PBS, pH = 7.4, Sigma-Aldrich, France) was added at a ratio of 2 mg sponge/mL PBS. The vial was incubated at 37 °C with orbital shaking at 80 rpm for 24 h, during which the scaffold was repeatedly withdrawn and put on an absorbent paper to remove the excess water for weighing at 0.5 h, then every hour for 6 h, and finally 24 h. The percentage of water uptake was determined according to the following formula:

(1) $$\%\;\mathrm{Water}\;\mathrm{uptake}\;=\;\left(\frac{W_{t}-W_{o}}{W_{o}}\right)\times 100$$

where W_t_ is the wet mass of sample, and W_o_ is its initial dry mass. All analyses were carried out in triplicate.

In Vitro Biodegradation

The in vitro degradation of CHT/PCD sponges was studied in the presence of lysozyme, which is the main enzyme that induces the degradation of CHT in the human body [39]. Briefly, the scaffolds were immersed in a solution of lysozyme (Sigma-Aldrich, Saint Quentin, France) in PBS at 0.5 mg/mL, and incubated at 37 °C with orbital shaking at 80 rpm. Sodium azide (Sigma-Aldrich, Saint Quentin, France) was added at a concentration of 0.5 mg/mL to prevent microbial contamination, and the lysozyme solution was renewed every 2 days. At predetermined times (1, 5, 14, and 21 days), the scaffolds were withdrawn, rinsed with distilled water and freeze-dried. The samples were weighed and the percentage of weight loss was determined according to the following formula:

(2) $$\%\;\mathrm{Weight}\;\mathrm{loss}\;=\;\frac{W_{t}-W_{o}}{W_{o}}\times 100$$

where W_t_ is the remaining mass of sample and W_o_ is its initial mass. All analyses were carried out in triplicate.

##### Mechanical Property

The mechanical compressive property of CHT/PCD sponge was evaluated in triplicate using a mechanical tester (Univert^®^, CellScale, Waterloo, ON, Canada). Sponges were cut into cylindrical samples (12 mm diameter × 11 mm height), and then, were fully hydrated by immersing them into a PBS solution for 2 h before the test. The system was equipped with a load cell of 10 N, and the test was performed up to 50% strain. The elastic modulus of compression (*E*) was determined as the slope of the linear elastic region in the stress–strain curve.

#### 2.2.3. Cytocompatibility

##### Cell Culture

Cytocompatibility was performed with two types of cells: bone-forming cells (MC3T3-E1) and angiogenic cells (HUVECs). MC3T3-E1 cells were cultured in Minimum Essential Medium (MEM-α, Gibco^®^, Thermo Fisher Scientific, France) supplemented with 10% fetal bovine serum (FBS, Gibco^®^, Thermo Fisher Scientific, Illkirch-Graffenstaden, France). HUVECs were cultured in Endothelial cell medium (ECM, PromoCell, Heidelberg, Germany) supplemented with 2.5% Endothelial Cell Growth Supplement (ECGS, PromoCell, Heidelberg, Germany) and 5% FBS. All cells were incubated in a humidified atmosphere, 5% CO_2_ at 37 °C (CB150/APT Line/Binder, LabExchange, Paris, France).

##### Cell Viability

First, CHT/PCD sponges were disinfected by immersing the samples in 1 mL of 70% ethanol (Fisher Chemical, Fischer Scientific, Illkirch-Graffenstaden, France) for 1 h at RT and 80 rpm. Then, the scaffolds were rinsed with a sterile Hanks Balanced Saline Solution (HBSS, Gibco^®^, Thermo Fischer Scientific, Illkirch-Graffenstaden, France) for 1.5 h at 37 °C and 80 rpm. Each scaffold was further immersed in the respective cell culture medium overnight, at 37 °C and 80 rpm. Finally, samples were put in a sterile Petri dish, ready for cell seeding.

MC3T3-E1 cells and HUVECs were recovered respectively by trypsinization from a cell culture dish and centrifuged. Then, the cells were seeded by depositing 30 μL cell suspension dropwise over the entire surface of the scaffold to reach a density of 3.5 × 10^3^ cells/mm^3^ and 5.3 × 10^3^ cells/mm^3^ for the MC3T3-E1 cells and HUVECs, respectively. Cell-seeded scaffolds were incubated for 2 h at 37 °C and 5% CO_2_ in order to allow the cells time to adhere to the scaffold, then 3.5 mL of the corresponding medium was added to the scaffold. The samples were incubated at 37 °C and 5% CO_2_ for 3 or 6 days. Cell culture medium was renewed every 3 days.

##### LIVE/DEAD^®^ Assay

At a predetermined time (3 or 6 days), the LIVE/DEAD^®^ kit (Thermo Fischer Scientific, Illkirch-Graffenstaden, France) was used to evaluated cell viability. In brief, the samples were carefully rinsed with 500 μL/well of HBSS for 3 min at 37 °C. Then a solution (500 μL) containing 2 μM Calcein and 1 μM Ethidium homodimer was added to each scaffold. After 30 min of incubation, protected from light and at room temperature, the scaffolds were placed in a µ-Dish 35 mm-high Glass Bottom dish (Ibidi GmbH, Gräfelfing, Germany), and the labeled cells were imaged using an LSM 710 laser-scanning confocal microscopy (Zeiss, Oberkochen, Germany). The wavelengths used were 494/517 nm for Calcein and 528/617 nm for the Ethidium homodimer.

#### 2.2.4. VEGF-Loading on CHT/PCD Sponges

Sterile CHT/PCD sponges (6 mm in diameter × 2–2.5 mm thick) were immersed in a sterile saline solution (NaCl 0.9%, Versylene^®^, Fresenius Kabi, Sèvres, France) overnight at 37 °C and 80 rpm, and then rinsed with ultrapure water. The scaffolds were frozen and freeze-dried (0.06 mbar, −53 °C).

An aqueous solution of VEGF (10 µg/mL) was diluted in PBS supplemented with 0.1% bovine serum albumin (BSA, Sigma-Aldrich, Saint Quentin, France) at different predetermined concentrations. Then, a loading method based on physical adsorption was applied. Briefly, thirty microliters of VEGF solution were added dropwise in each sponge, followed by an overnight incubation at 4 °C to ensure maximum physical adsorption of VEGF on the sponge. Loaded scaffolds were frozen at −20 °C and freeze-dried (0.06 mbar, −53 °C) for 24 h. Finally, VEGF-loaded scaffolds (20, 50, and 100 ng VEGF per sample) were stored at −20 °C until use.

#### 2.2.5. In Vitro VEGF Release 

VEGF-loaded scaffolds were put in a 24-well plate, then 1 mL of ECM medium supplemented with 0.5% FBS was added to each well, and the plate was incubated at 37 °C. The release medium was collected and entirely replaced with ECM medium supplemented with 0.5% FBS at selected points in time (1, 2, 3, 5, and 7 days). The amount of released VEGF was determined by the immunoenzymatic ELISA (n = 3/time point) sandwich method following the manufacturer’s instructions (PeproTech, Neuilly-sur-Seine, France). Finally, the absorbance reading at 405 nm was made in a Multiskan™ FC microplate spectrometer (Thermo Fischer Scientific, Illkirch-Graffenstaden, France).

#### 2.2.6. Bioactivity of Released VEGF from Sponges 

The bioactivity of VEGF released from CHT/PCD sponges was evaluated by its effect on the proliferation and migration of endothelial cells (HUVECs), which is the key step of the angiogenic process. The VEGF release experiment was undertaken by immersing the VEGF-loaded and non-loaded scaffolds in ECM medium supplemented with 0.1% FBS [15], and the VEGF release medium was collected at days 1, 2, 3, and 7 and stored at 4 °C until the use for following proliferation and migration assay.

##### HUVECs Proliferation Assay

Cells were seeded at a density of 3.5 × 10^3^ cells/well in a 96-well plate and incubated for 6 h at 37 °C. Then, the culture medium was replaced with ECM medium supplemented with 0.1% FBS and incubated overnight at 37 °C and 5% CO_2_ in a nutrient-deprived culture condition. After incubation, the nutrient-deficient medium was replaced with the VEGF release medium collected from the VEGF release process described above, and the plate was incubated for 48 h. A positive control (50 ng/mL VEGF) and a negative control (ECM medium supplemented with 0.1% FBS) were also tested. The proliferation of the cells was finally quantified by a fluorometric assay with AlamarBlue^®^ reagent (Gibco^®^, Thermo Fisher Scientific, Illkirch-Graffenstaden, France) [34].

##### HUVECs Migration Assay

The in vitro scratch assay, which imitates cell migration during wound healing in vivo, is an easy, low-cost and well-developed method to measure cell migration in vitro [40]. In short, HUVECs were seeded at a density of 5 × 10^4^ cells/well in a 4-well plate (Nunc™, Thermo Fischer Scientific, Illkirch-Graffenstaden, France), and incubated for 24 h until they formed a confluent monolayer. Then, a “wound” was formed by scraping an area of the cell monolayer using a universal pipette tip (200 µL, Thermo Fisher Scientific, Illkirch-Graffenstaden, France). Each well was then rinsed with ECM medium supplemented with 0.1% FBS and photographed using phase contrast microscopy on an inverted microscope (DM IL LED, Leica, Wetzlar, Germany) to record the initial wound. Five hundred microliters of VEGF release medium of each scaffold, positive control (50 ng/mL VEGF/ECM medium) or negative control (ECM medium with 0.1% FBS) was gently added to the cell monolayer and was incubated at 37 °C and 5% CO_2_ for 24 h [40].

After incubation, the medium of each well was removed and the cells were fixed and stained with 0.2% Crystal-violet solution. Wounds were photographed for recording the final wound shape. Initial and final wound sizes were determined using the software Image J (Version 1.50 26, Bethesda, MD, USA), and the percentages of reduction of the width of the wound with regard to the initial wound size, according to the following formula, were used to determine the migration capacity.

$$\%\;\mathrm{reduction}\;\mathrm{of}\;\mathrm{the}\;\mathrm{width}\;\mathrm{of}\;\mathrm{the}\;\mathrm{wound}\;=\;\frac{W_{t}-W_{o}}{W_{o}}\times 100,$$

where W_t_ is width of the final wound and W_o_ is width of the initial wound. Three independent experiments were carried out.

#### 2.2.7. Statistical Analysis

The mean and standard deviation (SD) of the quantitative data were calculated for all manipulations. Statistical analysis of data was carried out using the software program Prism (version 5, GraphPad Software, La Jolla, CA, USA). T-student test, one-way ANOVA and Tukey’s Post-Hoc Test were applied to the data in order to assess significant differences. The differences were considered statistically significant by *p*-value < 0.05.

## 3. Results and Discussion 

### 3.1. Characterization of Macroporous Hydrogels

Cylindrical CHT/PCD sponges were obtained after freeze-drying. From a macroscopic point of view, the cross-sections at different levels of the freeze-dried cylinder showed a shade of yellow-beige and a homogeneous porous feature (Figure 1B). After thermal treatment, a brownish tint appeared on the scaffolds (Figure 1C), which could be the response to the rearrangement of CHT chains and cross-linking of polymer chains: The formation of covalent amide bonds during the heat treatment [41]. Since the covalent bond is energetically stronger than an electrostatic bond, therefore, it could improve the stability of the scaffold [42], e.g., degrade much more slowly as described by Gauzit Amiel et al. [43].

#### 3.1.1. Microstructure

**Figure 2:**
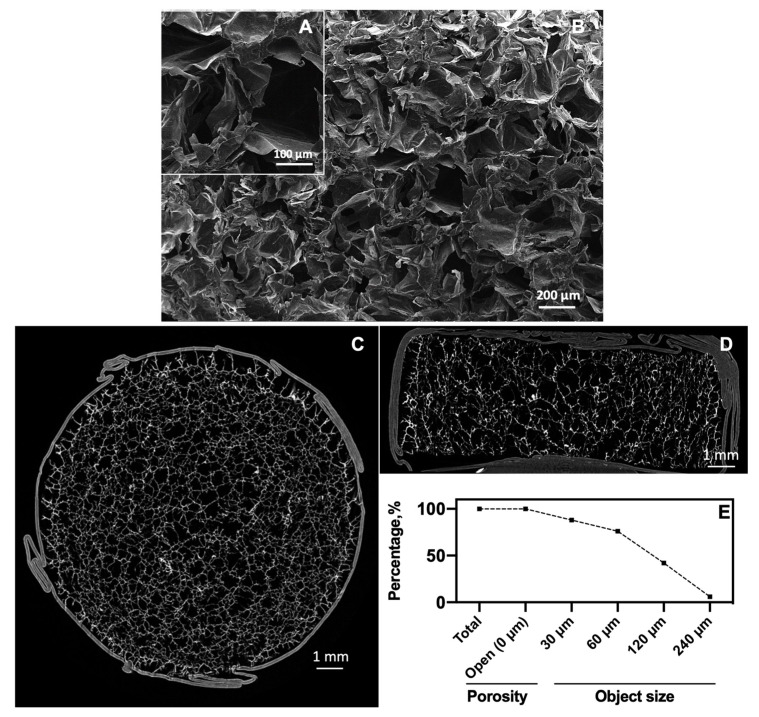
Analysis of the microstructure of the CHT/PCD sponge. SEM images of the cross-section of the scaffold: (**A**) At ×50 and (**B**) at ×100 magnification. µCT cross-section images: (**C**) Horizontal view, (**D**) vertical view (both images show the parafilm layer used around the samples), and (**E**) percentage of available interconnected porosity to allow passage of an object of defined size into the scaffold.

The SEM imaging (Figure 2A,B) gave a first visualization of the microstructure morphology of the cross-sections of the sponges: They were porous and seemed to be interconnected. A zoomed view (Figure 2A) of the sponges revealed that the walls of pores were thin and smooth.

The μCT analysis (Figure 2C,D) of the sponge confirmed an interconnected porosity with homogeneously distributed pores of different sizes in the scaffold. The quantitative analysis of the interconnectivity (Figure 2E) indicated that the sponge has a total open and accessible porosity of approximately 87.5% and an average pore size of 153 μm. In general, as the volume of the object increased, access to the interconnecting passages decreased. When setting the volume objects at 30, 60, 120 and 240 μm, the percentage of accessibility was 88%, 76%, 42%, and 6%, respectively. The scaffold had an average porosity accessibility of about 120 μm, i.e., objects of this size can enter in about 49% of the porosity of the sponge. In contrast, objects of about 200 μm were able to only penetrate 6% of the accessible pores in the scaffold. For scaffolds of BTE, the pore sizes between 100 and 350 μm (macropores) is regarded as important as they allow cell and tissue in-growth, and invasion of the vascular system; while pores <20 μm (micropores) can offer a larger surface area which could promote the adsorption of biomolecules [44,45]. Nevertheless, the interconnection of pores to different object sizes is also an important parameter for supporting cell migration and the diffusion of nutrients [7]. Our CHT/PCD sponge met the above requirements for porosity and interconnectivity for a BTE application.

#### 3.1.2. Water Uptake

The water absorption of the scaffolds is a very important parameter for the BTE. The initial absorption allows the increase of pore size, which facilitates invasion, adhesion and cell growth in the 3D scaffold [46]. Water uptake is also important for drug loading by absorption and for drug delivery by diffusion from sponges [31]. However, excessive swelling can lead to the stress in the surrounding tissues and reduce the mechanical strength of the scaffold due to a structural collapse [47,48].

**Figure 3:**
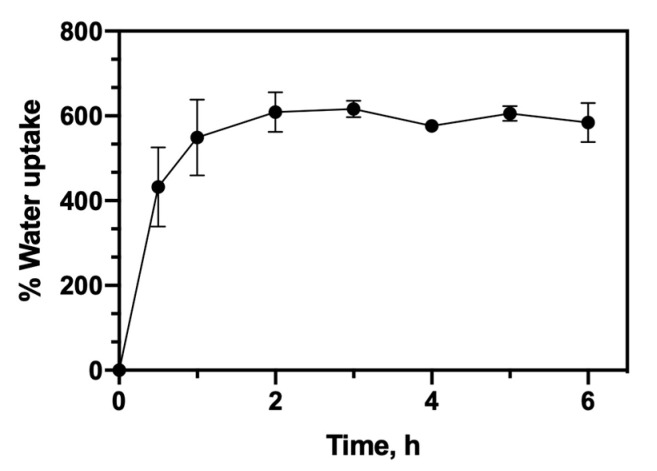
Water uptake profile of CHT/PCD sponge in PBS (pH 7.4) at 37 °C and 80 rpm. The test was carried out at least twice and each in triplicate (*n* = 6).

The water uptake kinetics of the CHT/PCD sponge in PBS is shown in Figure 3. In general, the studied scaffold reaches its maximum water absorption rate (about 600%) without any significant dimensional variation after one-hour immersion in PBS. Indeed, this rapid initial water absorption capacity (without excessive swelling) of CHT/PCD scaffolds would be advantageous for use in BTE. Thus, cells will be allowed to invade and grow inside the sponge due to this water uptake profile. Furthermore, CHT/PCD scaffolds kept the integrity of their structure in wet conditions during the test.

#### 3.1.3. Biodegradation

In order to mimic the in vivo conditions, the study of the biodegradation of the sponge was carried out using a solution of PBS enriched with 0.5 mg/mL of lysozyme. Lysozyme is the major enzyme that degrades CHT in the human body by cutting the glycosidic bonds between the polysaccharide units of CHT [49]. The study of Lončarević et al. [50] demonstrated the impact of lysozyme on degradation of chitosan compared to a lysozyme-free PBS solution, and concluded that the design of degradation experiments and the characterization of degradation behavior of chitosan-based scaffolds used as implants or drug delivery systems should be studied in lysozyme-PBS solution.

**Figure 4:**
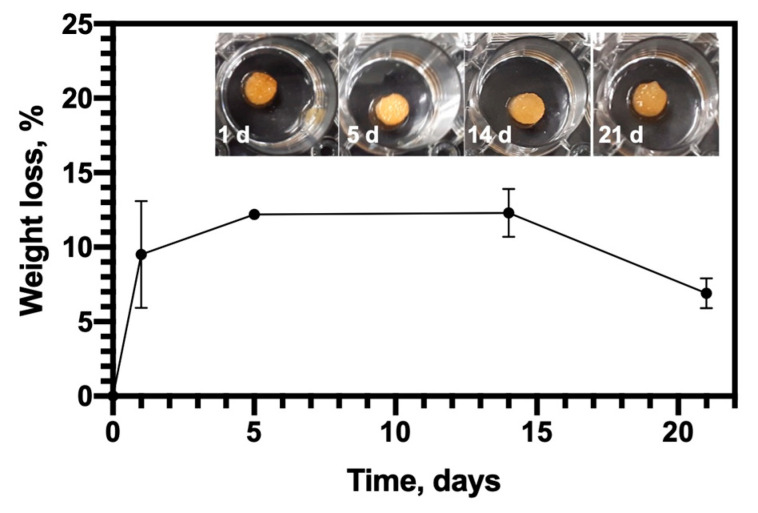
Biodegradation of CHT/PCD sponge (expressed in percentage of weight loss) in PBS (pH 7.4) supplemented with 0.5 mg/mL lysozyme at 37 °C and 80 rpm and representative images of scaffolds removed after each time point up to 21 days. The test was carried out at least twice and each in triplicate (*n* = 6).

The biodegradation of the sponge and their macroscopic aspects after different degradation times are shown in Figure 4, the CHT/PCD scaffold degraded gently up to 12% until the end of the test (21 days). It must be noted that the values of degradation with time were not significantly different. This stability may be due to heat treatment as it forms covalent bonds between CHT and PCD and inter-chain cross-linking of CHT [51]. Thus, CHT/PCD scaffold acquires a better stability, which is reflected in its slow biodegradation [43]. Indeed, such biodegradation profile of CHT/PCD sponges seems to be more adapted to support cell growth to generate neo-tissue, and could be resorbed at the same rate as the regeneration of the tissue.

#### 3.1.4. Mechanical Property

**Figure 5:**
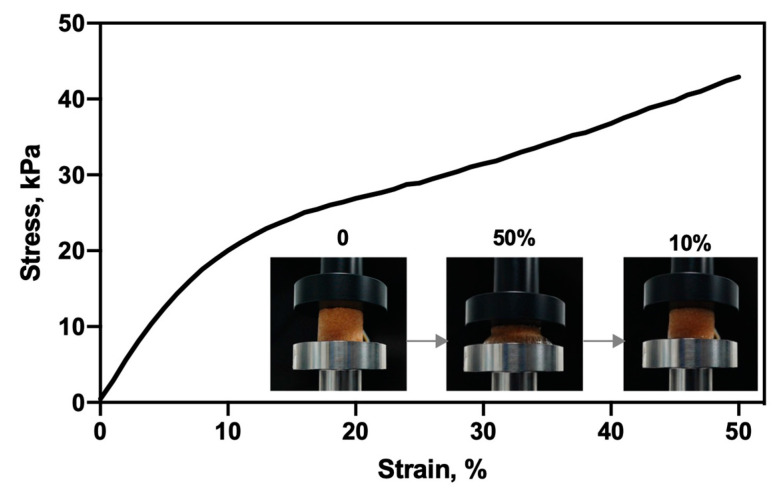
Compressive stress–strain curve of CHT/PCD sponge after immersion in PBS for 2 h, tested at room temperature. Representative images of sponge during the compression test. On the left, the scaffold at the beginning of the test; in the middle, scaffold compressed at 50% strain; on the right, scaffold recovered their shape at the end of the test (10% strain). The curve corresponds to the average values of three repetitions (*n* = 3).

The mechanical properties of biomaterials are important for its application as a scaffold for BTE. For this purpose, the hydrated sponge was evaluated in their mechanical behavior under compression. Compressive stress–strain curve of the CHT/PCD sponge is shown in Figure 5, and displays two characteristic regions of the elastic materials [52,53]: A linear elastic (between 1 and 10% strain) region, which exhibits a very rapid increase of stress at low strain; followed by a nearly flat region (“plateau”) which represents plastic deformation. For this sponge, the stress increases moderately to a high strain (up to 50%). The phase of “rupture” or “fracture” did not appear because the maximum strain was limited at 50% [52,53]. Based on the linear region of the stress–strain curve, the elastic modulus of compression (*E*) was calculated as 256 ± 4 kPa, which was 20 times higher than that of without thermal treatment (12 ± 1 kPa, see Figure S1). Clearly, thermal treatment improves the mechanical properties of sponge as reported by Gauzit Amiel et al. [43]. Moreover, the sponge demonstrated a total recovery after compression (50%) in the mechanical tester with a 10 N load cell (Figure 5).

Biodegradable polymer scaffolds often have much lower mechanical properties than bioceramic ones [2] and that of the cancellous bone (0.1–2 GPa) [54]. However, it has been reported that scaffolds with a Young’s modulus of 134 kPa can favor osteoblastic differentiation [55]. Kuo et al. [56] also reported a commercial gelatin-based sponge (Spongostan™) with a compression modulus of 170 ± 6 kPa, which is effective for promoting the differentiation of pre-osteoblast cell. In addition, the CHT/PCD sponge demonstrated an improved mechanical property (256 ± 4 kPa) compared to other scaffolds of the same category; thus, it is adaptable for the use in BTE.

### 3.2. Cytocompatibility

After the physicochemical characterization of the scaffold, the cellular response (cell viability) was assessed with two relevant types of cells: Pre-osteoblasts (MC3T3-E1) and endothelial cells (HUVECs).

**Figure 6:**
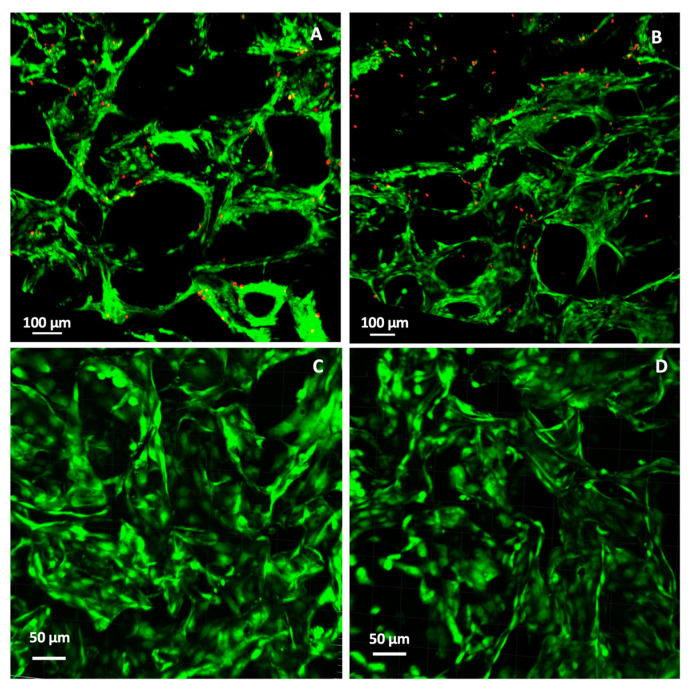
2D fluorescence confocal microscopy images of the LIVE/DEAD^®^ assays of (**A**,**B**) pre-osteoblast cells (MC3T3-E1) and (**C**,**D**) endothelial cells (HUVECs) in CHT/PCD sponge after (**A**–**C**) 3-day and (**B**–**D**) 6-day static culture. Green fluorescence: living cells; red fluorescence: dead cells.

The LIVE/DEAD^®^ assay differentially labels live and dead cells with fluorescent dyes in CHT/PCD sponges. The fluorescent microscopy images (Figure 6A,B) of MC3T3-E1 cells growing on the scaffold after 3 and 6 days showed that the scaffold was cell-colonized: Living cells were predominant and were well adhered to the walls of pores in the scaffold, which confirmed the good interaction of osteoblasts with CHT-based scaffolds [57] after 3 and 6 days of culture. These results demonstrate the good cell viability of pre-osteoblast cells into CHT/PCD sponge.

Similarly, HUVECs were also found to be firmly adhered and to have colonized the porous structure of the scaffolds after 3 and 6 days of culture (Figure 6C,D). Living cells were observed in very large numbers and dead cells were very rarely observed. Mohandas et al. [58] have also reported excellent adhesion to HUVECs cells on the surface of a hybrid sponge of CHT and hyaluronic acid. Therefore, such good interaction between HUVECs and CHT based sponge can support their application as scaffolds for BTE.

### 3.3. In Vitro VEGF Release 

**Figure 7:**
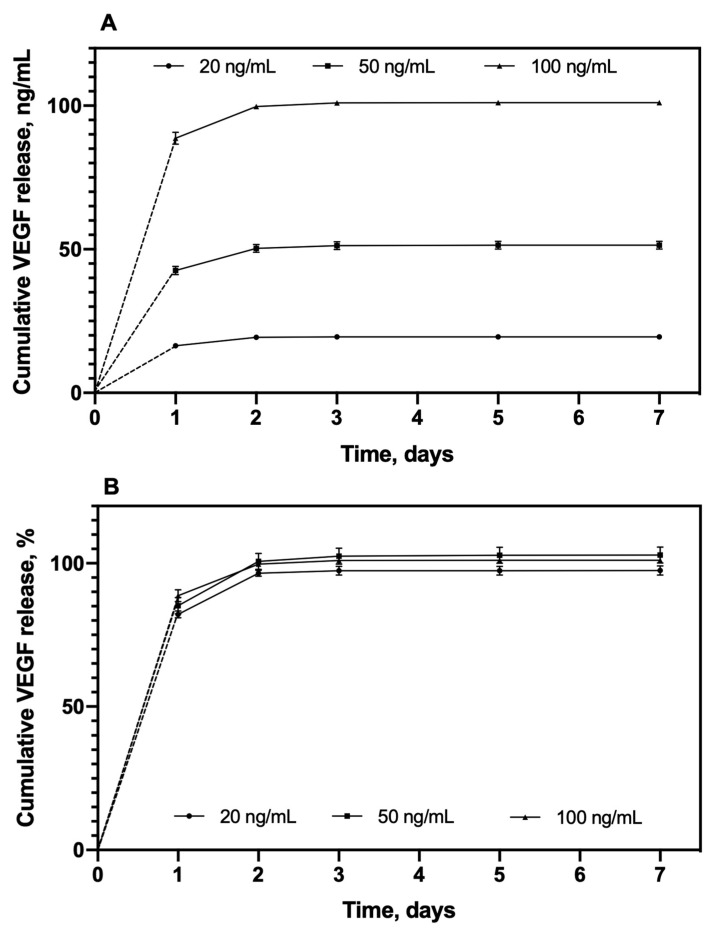
In vitro cumulative release profiles expressed (**A**) in ng/mL and (**B**) as percentage of CHT/PCD sponges loaded with 20, 50, and 100 ng VEGF in ECM culture medium supplemented with 0.5% FCS at 37 °C under static conditions. The test was repeated twice and each in triplicate (*n* = 6).

The release profile of VEGF-loaded sponges containing 20 ng, 50 ng, and 100 ng VEGF were evaluated in the ECM culture medium supplemented with 0.5% FCS (Figure 7). The three groups with different VEGF amounts displayed a similarly rapid release profile. Indeed, more than 85% of the initial loading amount released within the first day, then the amount of released VEGF reduced with time to reach (at 7 days) a cumulative release amount of 19.5 ± 0.3 ng, 51.5 ± 13.9 ng, and 101.1 ± 0.7 ng for sponges loaded with 20, 50, and 100 ng VEGF, respectively (Figure 7A). Therefore, the total amount of released VEGF was close to the initial loading amount, which means that the entire quantity of loaded VEGF (97.5 ± 1.6%, 102.9 ± 27.7%, and 101.1 ± 0.7% with respect to the initial loaded VEGF amount 20 ng, 50 ng, and 100 ng, respectively) was totally released (Figure 7B). Thus, the desirable amount of VEGF release (i.e., the required bioactive dose) could be easily adjusted by varying the amount of initial VEGF load. A study of further monitoring the VEGF release during the first day, revealed an initial “burst” of approximately 40% of the total quantity within the first half hour for all 3 concentration groups, followed by a moderate and sustained release for 24 h (Figure S2).

The release duration of VEGF from the scaffolds in our study was relatively short (almost 100% in 2 days), this could be explained by the mechanism of VEGF-loading into the scaffolds. Indeed, VEGF was loaded onto the sponges by the adsorption mechanism, that binds the protein with low-energy interaction, and the molecule is not chemically modified, compared to other mechanisms [19,59]. On the other hand, it is well-known that CD can form inclusion complexes with a large variety of low-molecular-mass drugs [60]. We have been able to demonstrate this interaction with ciprofloxacin [61], for example. Nevertheless, VEGF is a high-molecular-mass molecule, which is too large to enter into the hydrophobic cavity of CD. Thereby, VEGF can only interact through dipole interactions of van der Waals type, which can also explain the rapid molecule release in the aqueous medium by diffusion.

During bone healing, VEGF expression reaches a higher point at the earlier stages, followed by the expression of other GFs (i.e., BMPs) [12], and it is considered one of the pivotal regulator in angiogenesis [11]. In this context, Li et al. [62] have demonstrated that a rapid release of VEGF, especially in the critical phase (first 3 days), prompted the establishment of a very important local microcirculation for bone regeneration in mice. In order to determine if our VEGF-loaded sponges could release an appropriate amount of VEGF for inducing the formation of blood vessels, their bioactivity was evaluated as follows.

### 3.4. Bioactivity of Released VEGF

VEGF induces normal angiogenesis or aberrant vessels in a strictly dose-dependent fashion [63]. However, it revealed a discrete threshold in dosage separating normal and pathological angiogenesis. Only continuous expression of VEGF at the appropriate dose can produce normal and stable vascular growth while avoiding aberrant vasculature. After studying the kinetics of VEGF release, it is necessary to evaluate the intrinsic bioactivity or therapeutic potential of the released VEGF. Various in vitro techniques for evaluating the angiogenic properties in scaffolds are widely discussed in the literature [64,65]; we focused on evaluating the VEGF-induced proliferation and migration of HUVECs.

#### 3.4.1. HUVECs Proliferation Assay

**Figure 8:**
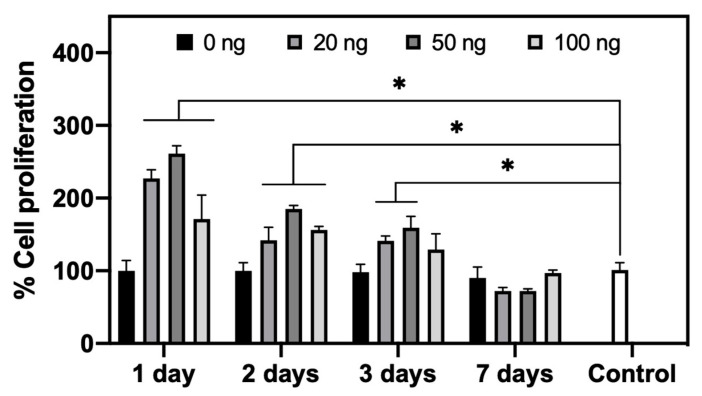
Bioactivity of released vascular endothelial growth factor (VEGF) in Endothelial cell medium (ECM) culture medium enriched with 0.1% FCS at 37 °C based on cell proliferation using Human Umbilical Vein Endothelial Cells (HUVECs) cells (* *p* < 0.05). The test was repeated twice and each in duplicate (*n* = 4).

Figure 8 shows the percentage of HUVEC proliferation after 48 h in contact with the release medium containing VEGF, compared to the control (culture medium with 0.1% FCS), which showed no pro-proliferation effects. A significantly higher cell proliferation (*p* < 0.05) than that found in the control can be observed until day 3 of release for the three groups (20, 50, or 100 ng VEGF load). However, there is no significant difference between groups (*p* > 0.05). It is clear that we did not observe a dose-dependent effect release VEGF even with significantly different concentrations between groups (Table 1). Moreover, with similar a concentration of VEGF (~ 0.23 ± 0.05 ng/mL), at day 3 the 20 ng group showed a pro-proliferation effect, while at day 7 the 100 ng group no longer influenced the proliferation of HUVECs. These results reveal that, a priori, under cellular culture conditions, the study of dose-response relationship of VEGF in vitro may vary from case to case. However, in our study, the concentration of VEGF at less than 0.23 ± 0.05 ng/mL (Table 1) seems to be the low threshold of an effective dose for the pro-proliferation effect of VEGF.

#### 3.4.2. HUVECs Migration Assay

**Figure 9:**
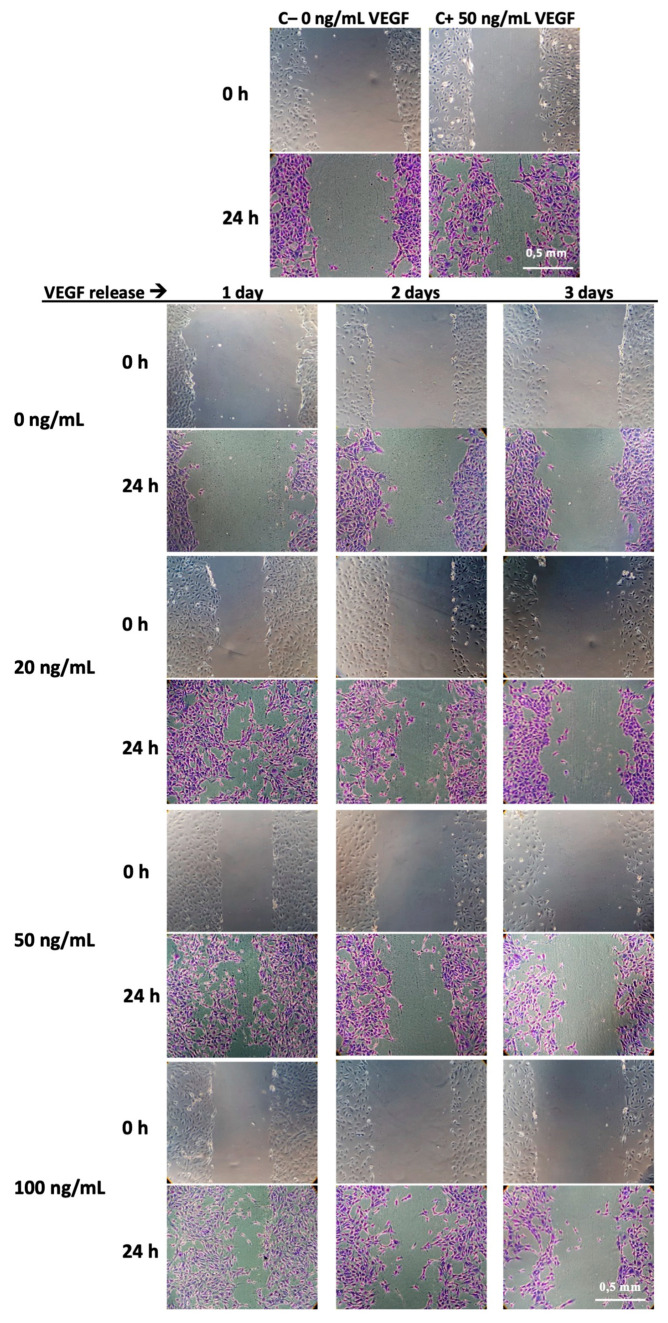
Representative images of “wound” shape in scratch wound migration assay, with HUVECs cell cultured in the VEGF release medium collected from 3-day release test of different VEGF-loaded sponges (0, 20, 50, and 100 ng VEGF/scaffold). The images represent the wound shape at its creation (0 h) and the healing by migration of HUVECs cells after 24 h. The test was repeated twice and each in duplicate (*n* = 4).

The scratch wound migration assay was performed only with VEGF release samples until the third day of release. Applying this technique, we evaluated the ability of the released VEGF to promote the “healing” of a wound made on a cell monolayer. The healing effect evidenced by this method normally encompasses cell migration and proliferation, but the latter can be minimized in this study by using a serum-poor medium (only 0.1% FCS) [64]. Figure 9 shows representative images of wound shape at the beginning of the test (0 h) and the healing by migration of HUVEC cells after 24 h. A cell culture medium without VEGF was used as negative control, as well as the release medium in contact with the non-loaded CHT/PCD scaffold. The cell culture medium enriched with 50 ng/mL of VEGF was used as positive control.

At first, the negative control (medium without VEGF) and the release medium from non-loaded-scaffold (0 ng/mL) did not show significant cell migration. In contrast, positive control (medium with 50 ng/mL) clearly showed an active cell migration and, therefore, a significant reduction in wound width. For all VEGF-loaded groups, the VEGF release medium of the first day showed a high pro-migration effect by clearly visible reduction of the width of the wounds (Figure 9). Afterwards, the VEGF release medium from the second or third day showed a markedly lower pro-migration effect. This reduction of bioactivity is consistent with the results found in the pro-proliferation assay, both related to the diminution of VEGF concentration in the medium (Table 1).

**Figure 10:**
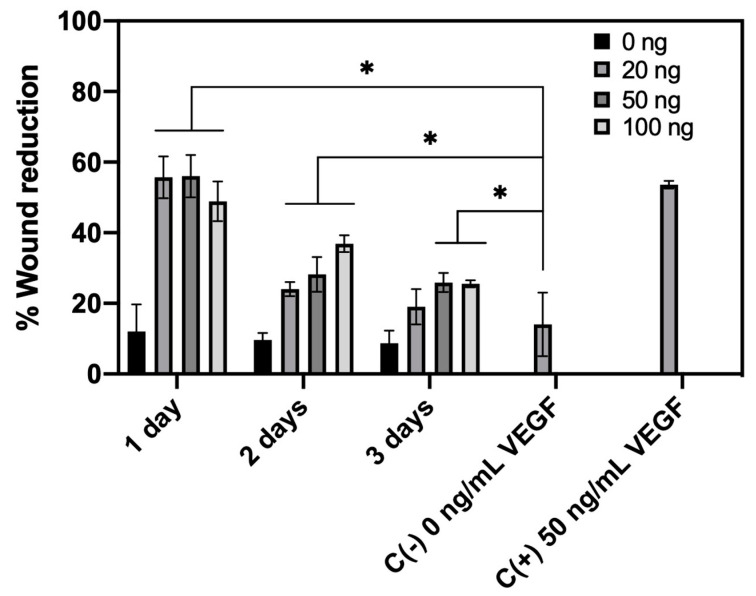
Quantitative analysis of the pro-migration effect of released VEGF expressed as a relative percentage of the wound gap reduction on a HUVEC monolayer after 24 h (* *p* < 0.05). The test was repeated twice and each in duplicate (*n* = 4).

The percentages of reduction of the injury, obtained by ImageJ and presented in Figure 10, confirm the above observation. Wound width reduction was approximately 55% for VEGF release medium of day 1 of all VEGF-loaded groups, which were significantly stronger (*p* < 0.05) than the negative control (15%) or non-loaded group (12%), and equivalent to that of positive control (52%). Then, for the VEGF release medium of all VEGF-loaded groups at day 2 and day 3, the wound reduction of the injury became less strong (about 30% and 20% respectively) than that of day 1 or the positive control. Despite this, the reduction was still superior (*p* < 0.05) than the negative control.

Regarding the dose-effect relationship, a lower concentration of released VEGF (less than 11 ng/mL VEGF in release medium of day 2 and 3) induced less significant wound healing than a high concentration of released VEGF (release medium of day 1). Then again, a higher VEGF concentration (84.26 ± 4.78 ng/mL VEGF in release medium of day 1 100 ng VEGF scaffold) (Table 1) did not show any significant differences (*p* > 0.05) from the positive control (50 ng/mL VEGF). Thus, even if the VEGF concentration is higher than 50 ng/mL, bioactivity is not increased. In addition, there is no significant difference (*p* > 0.05) between the VEGF-loaded groups, although the concentrations of released VEGF were quite different from each other (18.5 ± 0.80 ng/mL, 43.17 ± 3.00 ng/mL, and 84.26 ± 4.78 ng/mL, respectively). These results show that, under the applied test conditions, the dose-dependent effect of VEGF on the migration of HUVECs may be limited; beyond a certain threshold (~18 ng/mL), the pro-migration effect of VEGF may no longer increase with a rise in concentration.

Therefore, these two analyses demonstrated that bioactivity of VEGF was maintained after release, notwithstanding all preparation stages of scaffold biofunctionalization. At this point, it is not yet clear which concentration of VEGF should be chosen.

## 4. Conclusions

This study demonstrated that the CHT/PCD sponge is an adequate vehicle for VEGF stability and release. The characterization of this scaffold in terms of porosity, water uptake, degradation and cytocompatibility showed that it will be suitable for BTE application. In vitro bioactivity of the VEGF released from sponges was evidenced by the promotion of cell migration and proliferation, the main properties of scaffold vascularization. On the other hand, the addition of bioceramics, e.g., hydroxyapatite, will be performed for further improving the mechanical properties of sponge and prolonging the VEGF release. Finally, in vivo analysis will be necessary for determinate the ideal VEGF concentration.

## Figures and Tables

**Table 1:** Concentration of released VEGF within 7 days in ECM culture medium enriched with 0.1% FCS at 37 °C.

Release	VEGF-Loaded		
Days	20 ng	50 ng	100 ng
	Concentration (ng/mL)	Concentration (ng/mL)	Concentration (ng/mL)
**1**	18.5 ± 0.80	43.17 ± 3.00	84.26 ± 4.78
**2**	1.95 ± 0.06	6.12 ± 2.60	11.03 ± 0.99
**3**	0.23 ± 0.05	0.92 ± 0.53	1.82 ± 1.27
**7**	0.05 ± 0.00	0.10 ± 0.15	0.24 ± 0.03

## Supplementary Materials

The following are available online at https://www.mdpi.com/1999-4923/12/9/784/s1, Figure S1: Compressive stress–strain curve of CHT/PCD sponge without thermal treatment after immersion in PBS for 2 h, tested at room temperature. Representative images of sponge during the compression test. On the left, the scaffold at the beginning of the test; in the middle, scaffold compressed at 50% strain; on the right, scaffold at the end of the test (10% strain). The curve corresponds to the average values of three repetitions (*n* = 3). Figure S2: In vitro cumulative release profiles at the firsts days expressed (A) in ng/mL and (B) as percentage of CHT/PCD sponges loaded with 20, 50 and 100 ng VEGF in ECM culture medium supplemented with 0.5% FCS at 37 °C under static conditions. The curves correspond to the average values of three repetitions (*n* = 3).

## References

[1] Lindahl A., Brittberg M., Gibbs D., Dawson J.I., Kanczler J., Black C., Tare R., Oreffo R.O.C. (2015). Cartilage and Bone Regeneration. Tissue Engineering.

[2] Amini A.R., Laurencin C.T., Nukavarapu S.P. (2012). Bone tissue engineering: Recent advances and challenges. Crit. Rev. Biomed. Eng..

[3] Mehta M., Schmidt-Bleek K., Duda G.N., Mooney D.J. (2012). Biomaterial delivery of morphogens to mimic the natural healing cascade in bone. Adv. Drug Deliv. Rev..

[4] Wang W., Yeung K.W.K. (2017). Bone grafts and biomaterials substitutes for bone defect repair: A review. Bioact. Mater..

[5] Shakya A.K., Kandalam U. (2017). Three-Dimensional macroporous materials for tissue engineering of craniofacial bone. Br. J. Oral Maxillofac. Surg..

[6] Tollemar V., Collier Z.J., Mohammed M.K., Lee M.J., Ameer G.A., Reid R.R. (2016). Stem cells, growth factors and scaffolds in craniofacial regenerative medicine. Genes Dis..

[7] Akter F., Ibanez J. (2016). Bone and Cartilage Tissue Engineering. Tissue Engineering Made Easy.

[8] Lin Y., Huang S., Zou R., Gao X., Ruan J., Weir M.D., Reynolds M.A., Qin W., Chang X., Fu H. (2019). Calcium phosphate cement scaffold with stem cell co-culture and prevascularization for dental and craniofacial bone tissue engineering. Dent. Mater..

[9] Boontheekul T., Mooney D.J. (2003). Protein-based signaling systems in tissue engineering. Curr. Opin. Biotechnol..

[10] Fu J., Wang D.-A. (2018). In Situ Organ-Specific Vascularization in Tissue Engineering. Trends Biotechnol..

[11] De Rosa L., Di Stasi R., D’Andrea L.D. (2018). Pro-angiogenic peptides in biomedicine. Arch. Biochem. Biophys..

[12] Hu K., Olsen B.R. (2016). The roles of vascular endothelial growth factor in bone repair and regeneration. Bone.

[13] Beamer B., Hettrich C., Lane J. (2009). Vascular Endothelial Growth Factor: An Essential Component of Angiogenesis and Fracture Healing. HSS J..

[14] Carulli C., Innocenti M., Brandi M.L. (2013). Bone Vascularization in Normal and Disease Conditions. Front. Endocrinol..

[15] De Riva B., Nowak C., Sánchez E., Hernández A., Schulz-Siegmund M., Pec M.K., Delgado A., Évora C. (2009). VEGF-Controlled release within a bone defect from alginate/chitosan/PLA-H scaffolds. Eur. J. Pharm. Biopharm..

[16] Farokhi M., Mottaghitalab F., Shokrgozar M.A., Ai J., Hadjati J., Azami M. (2014). Bio-Hybrid silk fibroin/calcium phosphate/PLGA nanocomposite scaffold to control the delivery of vascular endothelial growth factor. Mater. Sci. Eng. C.

[17] Echave M.C., Pimenta-Lopes C., Pedraz J.L., Mehrali M., Dolatshahi-Pirouz A., Ventura F., Orive G. (2019). Enzymatic crosslinked gelatin 3D scaffolds for bone tissue engineering. Int. J. Pharm..

[18] Dou D.D., Zhou G., Liu H.W., Zhang J., Liu M.L., Xiao X.F., Fei J.J., Guan X.L., Fan Y.B. (2019). Sequential releasing of VEGF and BMP-2 in hydroxyapatite collagen scaffolds for bone tissue engineering: Design and characterization. Int. J. Boil. Macromol..

[19] Almubarak S., Nethercott H., Freeberg M., Beaudon C., Jha A., Jackson W., Marcucio R., Miclau T., Healy K., Bahney C. (2016). Tissue engineering strategies for promoting vascularized bone regeneration. Bone.

[20] Chen Z., Zhang Z., Ma X., Duan Z., Hui J., Zhu C., Zhang D., Fan D., Shang L., Chen F. (2019). Newly Designed Human-Like Collagen to Maximize Sensitive Release of BMP-2 for Remarkable Repairing of Bone Defects. Biomolecules.

[21] Claaßen C., Sewald L., Tovar G.E.M., Borchers K. (2017). Controlled Release of Vascular Endothelial Growth Factor from Heparin-Functionalized Gelatin Type A and Albumin Hydrogels. Gels.

[22] Suliman S., Xing Z., Wu X., Xue Y., Pedersen T.O., Sun Y., Døskeland A.P., Nickel J., Waag T., Lygre H. (2015). Release and bioactivity of bone morphogenetic protein-2 are affected by scaffold binding techniques in vitro and in vivo. J. Control. Release.

[23] Chiu L.L.Y., Radisic M. (2010). Scaffolds with covalently immobilized VEGF and Angiopoietin-1 for vascularization of engineered tissues. Biomaterials.

[24] Alarçin E., Lee T.Y., Karuthedom S., Mohammadi M., Brennan M.A., Lee D.H., Marrella A., Zhang J., Syla D., Zhang Y.S. (2018). Injectable shear-Thinning hydrogels for delivering osteogenic and angiogenic cells and growth factors. Biomater. Sci..

[25] Behr B., Sorkin M., Lehnhardt M., Renda A., Longaker M.T., Quarto N. (2012). A Comparative Analysis of the Osteogenic Effects of BMP-2, FGF-2, and VEGFA in a Calvarial Defect Model. Tissue Eng. Part A.

[26] Amirian J., Linh N.T.B., Min Y.K., Lee B.-T. (2014). The effect of BMP-2 and VEGF loading of gelatin-pectin-BCP scaffolds to enhance osteoblast proliferation. J. Appl. Polym. Sci..

[27] Dinu M.V., Dragan E.S., Thakur V.K., Thakur M.K. (2018). Macroporous Hydrogels: Preparation, Properties, and Applications. Hydrogels.

[28] Flégeau K., Pace R., Gautier H., Rethore G., Guicheux J., Le Visage C., Weiss P. (2017). Toward the development of biomimetic injectable and macroporous biohydrogels for regenerative medicine. Adv. Colloid Interface Sci..

[29] Berretta J., Bumgardner J.D., Jennings J.A. (2017). Lyophilized chitosan sponges. Chitosan Based Biomaterials, Volume 1.

[30] Bai X., Gao M., Syed S., Zhuang J., Xu X., Zhang X.-Q. (2018). Bioactive hydrogels for bone regeneration. Bioact. Mater..

[31] Bhattarai N., Gunn J., Zhang M. (2010). Chitosan-Based hydrogels for controlled, localized drug delivery. Adv. Drug Deliv. Rev..

[32] Yilmaz Atay H., Jana S. (2019). Antibacterial Activity of Chitosan-Based Systems. Functional Chitosan.

[33] Flores C., Lopez M., Tabary N., Neut C., Chai F., Betbeder D., Herkt C., Cazaux F., Gaucher V., Martel B. (2017). Preparation and characterization of novel chitosan and β-Cyclodextrin polymer sponges for wound dressing applications. Carbohydr. Polym..

[34] Palomino-Durand C., Lopez M., Cazaux F., Martel B., Blanchemain N., Chai F. (2019). Influence of the Soluble⁻Insoluble Ratios of Cyclodextrins Polymers on the Viscoelastic Properties of Injectable Chitosan⁻Based Hydrogels for Biomedical Application. Polymers.

[35] Martel B., Ruffin D., Weltrowski M., Lekchiri Y., Morcellet M. (2005). Water-Soluble polymers and gels from the polycondensation between cyclodextrins and poly(carboxylic acid)s: A study of the preparation parameters. J. Appl. Polym. Sci..

[36] Garcia-Fernandez M.J., Tabary N., Chai F., Cazaux F., Blanchemain N., Flament M.P., Martel B. (2016). New multifunctional pharmaceutical excipient in tablet formulation based on citric acid-cyclodextrin polymer. Int. J. Pharm..

[37] Mogrovejo-Valdivia A., Rahmouni O., Tabary N., Maton M., Neut C., Martel B., Blanchemain N. (2018). In vitro evaluation of drug release and antibacterial activity of a silver-Loaded wound dressing coated with a multilayer system. Int. J. Pharm..

[38] Lopez-Heredia M.A., Sariibrahimoglu K., Yang W., Bohner M., Yamashita D., Kunstar A., Van Apeldoorn A.A., Bronkhorst E.M., Félix Lanao R.P., Leeuwenburgh S.C.G. (2012). Influence of the pore generator on the evolution of the mechanical properties and the porosity and interconnectivity of a calcium phosphate cement. Acta Biomater..

[39] Kean T., Thanou M. (2010). Biodegradation, biodistribution and toxicity of chitosan. Adv. Drug Deliv. Rev..

[40] Liang C.-C., Park A.Y., Guan J.-L. (2007). In vitro scratch assay: A convenient and inexpensive method for analysis of cell migration in vitro. Nat. Protoc..

[41] Bernabe P., Peniche C., Argüelles-Monal W. (2005). Swelling behavior of chitosan/pectin polyelectrolyte complex membranes. Effect of thermal cross-linking. Polym. Bull..

[42] Ji C., Shi J. (2013). Thermal-Crosslinked porous chitosan scaffolds for soft tissue engineering applications. Mater. Sci. Eng. C.

[43] Turnbull G., Clarke J., Picard F., Riches P., Jia L., Han F., Li B., Shu W. (2018). 3D bioactive composite scaffolds for bone tissue engineering. Bioact. Mater..

[44] Preethi Soundarya S., Haritha Menon A., Viji Chandran S., Selvamurugan N. (2018). Bone tissue engineering: Scaffold preparation using chitosan and other biomaterials with different design and fabrication techniques. Int. J. Boil. Macromol..

[45] Felfel R., Gideon-Adeniyi M.J., Zakir Hossain K.M., Roberts G.A.F., Grant D.M. (2019). Structural, mechanical and swelling characteristics of 3D scaffolds from chitosan-agarose blends. Carbohydr. Polym..

[46] Niranjan R.M., Koushik C., Saravanan S., Moorthi A., Vairamani M., Selvamurugan N. (2013). A novel injectable temperature-sensitive zinc doped chitosan/β-glycerophosphate hydrogel for bone tissue engineering. Int. J. Boil. Macromol..

[47] Singh B.N., Veeresh V., Mallick S.P., Jain Y., Sinha S., Rastogi A., Srivastava P. (2019). Design and evaluation of chitosan/chondroitin sulfate/nano-bioglass based composite scaffold for bone tissue engineering. Int. J. Boil. Macromol..

[48] Jennings J.A. (2017). Controlling chitosan degradation properties in vitro and in vivo. Chitosan Based Biomaterials, Volume 1.

[49] Lončarević A., Ivanković M., Rogina A. (2017). Lysozyme-Induced Degradation of Chitosan: The Characterisation of Degraded Chitosan Scaffolds. J. Tissue Repair Regen..

[50] Shamekhi M.A., Rabiee A., Mirzadeh H., Mahdavi H., Mohebbi-Kalhori D., Baghaban Eslaminejad M. (2017). Fabrication and characterization of hydrothermal cross-linked chitosan porous scaffolds for cartilage tissue engineering applications. Mater. Sci. Eng. C.

[51] Gil E.S., Kluge J.A., Rockwood D.N., Rajkhowa R., Wang L., Wang X., Kaplan D.L. (2011). Mechanical improvements to reinforced porous silk scaffolds. J. Biomed. Mater. Res. Part A.

[52] Seda Tığlı R., Karakeçili A., Gümüşderelioğlu M. (2007). In vitro characterization of chitosan scaffolds: Influence of composition and deacetylation degree. J. Mater. Sci. Mater. Electron..

[53] Amiel A.G., Palomino-Durand C., Maton M., Lopez M., Cazaux F., Chai F., Neut C., Foligné B., Martel B., Blanchemain N. (2020). Designed sponges based on chitosan and cyclodextrin polymer for a local release of ciprofloxacin in diabetic foot infections. Int. J. Pharm..

[54] Bose S., Roy M., Bandyopadhyay A. (2012). Recent advances in bone tissue engineering scaffolds. Trends Biotechnol..

[55] Zhang T., Lin S., Shao X., Zhang Q., Xue C., Zhang S., Lin Y., Zhu B., Cai X. (2017). Effect of matrix stiffness on osteoblast functionalization. Cell Prolif..

[56] Kuo Z.-K., Lai P.-L., Toh E.K.-W., Weng C.-H., Tseng H.-W., Chang P.-Z., Chen C.-C., Cheng C.-M. (2016). Osteogenic differentiation of preosteoblasts on a hemostatic gelatin sponge. Sci. Rep..

[57] Amaral I.F., Sampaio P., Barbosa M.A. (2005). Three-dimensional culture of human osteoblastic cells in chitosan sponges: The effect of the degree of acetylation. J. Biomed. Mater. Res. Part A.

[58] Mohandas A., Anisha B.S., Chennazhi K.P., Jayakumar R. (2015). Chitosan–hyaluronic acid/VEGF loaded fibrin nanoparticles composite sponges for enhancing angiogenesis in wounds. Colloids Surf. B: Biointerfaces.

[59] Chen F.-M., Zhang M., Wu Z.-F. (2010). Toward delivery of multiple growth factors in tissue engineering. Biomaterials.

[60] Davis M.E., Brewster M.E. (2004). Cyclodextrin-Based pharmaceutics: Past, present and future. Nat. Rev. Drug Discov..

[61] Blanchemain N., Karrout Y., Tabary N., Bria M., Neut C., Hildebrand H.F., Siepmann J., Martel B. (2012). Comparative study of vascular prostheses coated with polycyclodextrins for controlled ciprofloxacin release. Carbohydr. Polym..

[62] Li B., Wang H., Zhou G., Zhang J., Su X., Huang Z., Li Q., Wu Z., Qiu G. (2017). VEGF-Loaded biomimetic scaffolds: A promising approach to improve angiogenesis and osteogenesis in an ischemic environment. RSC Adv..

[63] Ozawa C.R., Banfi A., Glazer N.L., Thurston G., Springer M.L., Kraft P.E., McDonald D.M., Blau H.M. (2004). Microenvironmental VEGF concentration, not total dose, determines a threshold between normal and aberrant angiogenesis. J. Clin. Investig..

[64] Liu W.C., Chen S., Zheng L., Qin L. (2017). Angiogenesis Assays for the Evaluation of Angiogenic Properties of Orthopaedic Biomaterials—A General Review. Adv. Healthc. Mater..

[65] Irvin M.W., Zijlstra A., Wikswo J.P., Pozzi A. (2014). Techniques and assays for the study of angiogenesis. Exp. Boil. Med..

