# Supplementary material for: Chitosan/Polycyclodextrin (CHT/PCD)-Based Sponges Delivering VEGF to Enhance Angiogenesis for Bone Regeneration
Source: Pharmaceutics. 2020 Aug 19;12(9):784. doi: 10.3390/pharmaceutics12090784 (PMC7557476; doi:10.3390/pharmaceutics12090784)
Supplement: Supplementary file 1 [file pharmaceutics-12-00784-s001.pdf]

pharmaceutics

MDPI

Supplementary Materials: Chitosan/Polycyclodextrin (CHT/PCD)-Based Sponges Delivering VEGF to Enhance Angiogenesis for Bone Regeneration

Carla Palomino-Durand, Marco Lopez, Pierre Marchandise, Bernard Martel, Nicolas Blanchemain and Feng Chai

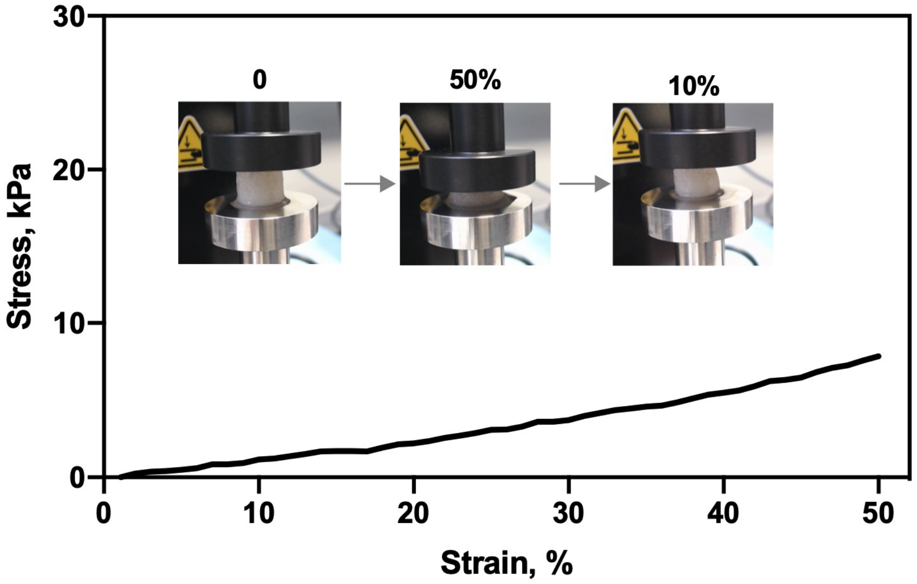

Figure S1. Compressive stress–strain curve of CHT/PCD sponge without thermal treatment after immersion in PBS for 2 h, tested at room temperature. Representative images of sponge during the compression test. On the left, the scaffold at the beginning of the test; in the middle, scaffold compressed at 50% strain; on the right, scaffold at the end of the test (10% strain). The curve corresponds to the average values of three repetitions ($n = 3$).

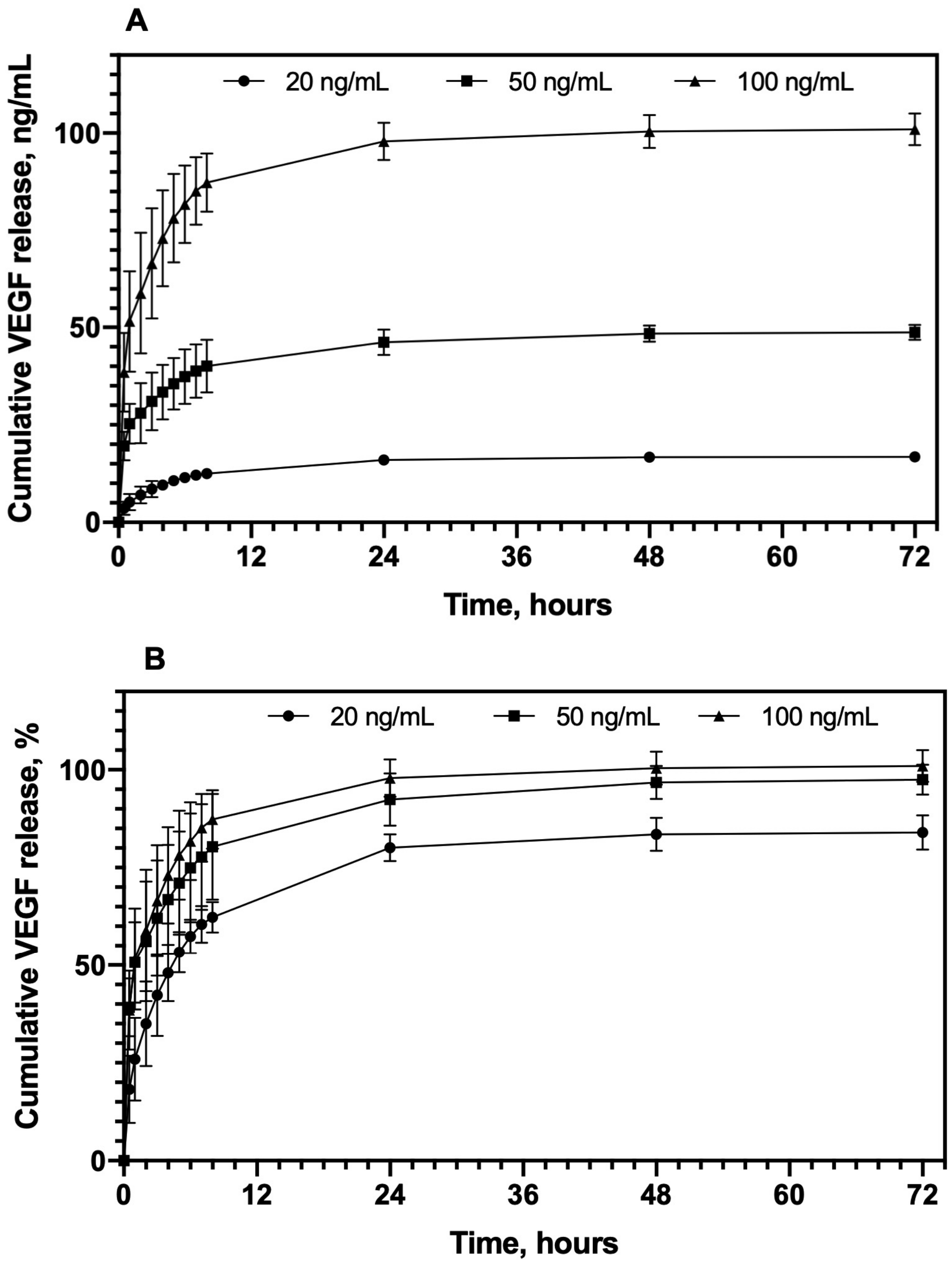

Figure S2. In vitro cumulative release profiles at the firsts days expressed (**A**) in ng/mL and (**B**) as percentage of CHT/PCD sponges loaded with 20, 50 and 100 ng VEGF in ECM culture medium supplemented with 0.5% FCS at 37 °C under static conditions. The curves correspond to the average values of three repetitions (*n* = 3).
